# Implementing NICE guidelines for the psychological treatment of depression and anxiety disorders: The IAPT experience

**DOI:** 10.3109/09540261.2011.606803

**Published:** 2011-10-25

**Authors:** David M Clark

**Affiliations:** University of Oxford, UK

## Abstract

The Improving Access to Psychological Therapies (IAPT) programme is a large-scale initiative that aims to greatly increase the availability of NICE recommended psychological treatment for depression and anxiety disorders within the National Health Service in England. This article describes the background to the programme, the arguments on which it is based, the therapist training scheme, the clinical service model, and a summary of progress to date. At mid-point in a national roll-out of the programme progress is generally in line with expectation, and a large number of people who would not otherwise have had the opportunity to receive evidence-based psychological treatment have accessed, and benefited from, the new IAPT services. Planned future developments and challenges for the programme are briefly described.

## Introduction

On World Mental Health Day in October 2007 the UK government announced a large-scale initiative for Improving Access to Psychological Therapies (IAPT) for depression and anxiety disorders within the English National Health Service (NHS). Between 2008 and 2011 at least 3,600 new psychological therapists will have been trained and employed in new IAPT clinical services offering the evidence-based psychological therapies that are recommended by the National Institute for Health and Clinical Excellence (NICE). A further cohort of around 2,400 new psychological therapists should be trained between 2011 and 2014, so that the services will have sufficient therapist capacity to offer treatment to at least 15% of people in the community with depression and/or anxiety disorders. The training follows national curricula and initially particularly focused on cognitive behavioural therapy (CBT), as this was where the manpower shortage was considered greatest. As the programme matures, training in other NICE recommended treatments for depression is also being made available. The clinical and other outcomes of patients who access the services are carefully monitored. This article describes the background to the programme, provides an overview of the training initiative and clinical service model, presents a summary of progress to date (early 2011), and anticipates future developments.

## Motivating circumstances

The IAPT programme had its roots in a wide range of clinical and policy developments. However, two developments deserve particular mention. First, starting in 2004, NICE systematically reviewed the evidence for the effectiveness of a variety of interventions for depression and anxiety disorders. These reviews led to the publication of a series of clinical guidelines (NICE, 2004a, 2004b, 2005a, 2005b, 2006, 2009a, 2009b, 2011) that strongly support the use of certain psychological therapies. CBT is recommended for depression and all the anxiety disorders. Some other therapies (interpersonal psychotherapy, behavioural couples therapy, counselling, brief dynamic therapy) are also recommended (with varying indications) for depression, but not for anxiety disorders. In the light of evidence that some individuals respond well to ‘low-intensity ’ interventions (such as guided self-help and computerized CBT) NICE also advocates a stepped-care approach to the delivery of psychological therapies in mild to moderate depression and some anxiety disorders. In moderate to severe depression and in some other anxiety disorders (such as post-traumatic stress disorder) low-intensity interventions are not recommended and instead it is suggested that patients should at once be offered ‘high-intensity ’ face-to-face psychological therapy. Table I summarizes the current NICE recommendations.

**Table I:** Summary of NICE's recommendations for the psychological treatment of depression and anxiety disorders.

Place in stepped-care service	Disorder	Recommended intervention
Step 3: High-intensity service (Primarily weekly, face-to-face, one-to-one sessions with a suitably trained therapist. In some disorders, such as depression, CBT can also be delivered effectively to small groups of patients. Behavioural couples therapy naturally involves the therapist, the depressed client and his/her partner)	Depression: moderate to severe	CBT or IPT^a^, each with medication
	Depression: mild to moderate	CBT or IPT^a^
		Behavioural activation (BA)^a^,^b^
		Behavioural couples therapy (if the patient has a partner, the relationship is considered to be contributing to the maintenance of the depression, and both parties wish to work together in therapy)
		Counselling a or short-term psychodynamic therapy^a^ (consider if patient has declined CBT, IPT, BA, or behavioural couples therapy)
	Panic disorder	CBT
	Generalized anxiety disorder (GAD)	CBT
	Post-traumatic stress disorder (PTSD)	CBT, EMDR
	Social phobia	CBT
	Obsessive - compulsive disorder (OCD)	CBT
Step 2: Low-intensity service (Less intensive clinician input than the high intensity service. Patients are typically encouraged to work through some form of self-help programme with frequent, brief guidance and encouragement from a PWP who acts as a coach)	Depression	Guided self-help based on CBT, cCBT, behavioural activation, structured physical activity
	Panic disorder	Self-help based on CBT, cCBT
	GAD	Self-help based on CBT, psycho-educational groups, computerized CBT
	PTSD	n/a^c^
	Social phobia	n/a
	OCD	Guided self-help based on CBT
Step 1: Primary care	Moderate to severe depression with a chronic physical health problem	Collaborative care (consider if depression has not responded to initial course of high intensity intervention and/or medication)

PWP, Psychological wellbeing practitioner; CBT, cognitive behavioural therapy; cCBT, computerized cognitive behavioural therapy; IPT, interpersonal therapy; EMDR, eye movement desensitization reprocessing therapy (considered by many to be a form of CBT); Behavioural activation is a variant of CBT; Active monitoring includes careful monitoring of symptoms, psycho-education about the disorder and sleep hygiene advice.

NICE has not yet issued guidance on the treatment of social phobia. However, there is a substantial body of evidence supporting the effectiveness of high-intensity CBT. Low intensity versions of CBT are being developed by several groups around the world and it seems likely that they will play a useful role in the future.

a NICE's recent (NICE, 2009a, 2009b) updates on the treatment of depression come in two parts: recommendations for the treatment of ‘depression’ and recommendations for the treatment of ‘depression in adults with a chronic physical health problem’. The two guidelines are very similar. However, it should be noted that the ‘depression with a physical health problem’ guideline does not recommend IPT, behavioural activation, counselling or brief dynamic therapy as high-intensity interventions.

b Although the recent update of the NICE guideline for depression (NICE, 2009a) recommends behavioural activation for the treatment of mild to moderate depression, it notes that the evidence base is not as strong as for CBT or IPT.

c NICE does not recommend any low-intensity interventions for PTSD and recommends that you do NOT offer psychological debriefing.

In the second development, economists and clinical researchers combined resources to argue that an increase in access to psychological therapies would largely pay for itself by reducing other depression- and anxiety-related public costs (welfare benefits and medical costs) and increasing revenues (taxes from return to work, increased productivity, etc.). This argument was advanced in academic articles (e.g. Layard *et al.*, 2007), but also in the more populist pamphlets such as *The Depression Report* (Layard *et al.*, 2006) and *We need to Talk* (Mind, 2010) (a report sponsored by numerous mental health and other charities). The latter were widely distributed to the public and to policy makers. For example, *The Depression Report* was included in every copy of a national newspaper (the *Observer* newspaper) on Sunday 18 June 2006.

The UK Government was receptive to the recommendations of NICE and to the broader arguments advanced in *The Depression Report* and elsewhere. A general political commitment to increase the availability of evidence-based psychological treatments was secured in 2005. However, before any decisions about the scale and form of the increase could be established, the government wisely decided to fund two pilot projects that would test whether the outcomes that one would expect from implementing NICE guidelines could be achieved in practice if a local area was given increased funding to recruit and deploy additional psychological therapists.

## Doncaster and Newham demonstration sites

In 2006 the National Health Service (NHS) in England comprised 154 primary care trusts (PCTs), each of which had responsibility for the health care of its local population. Two PCTs (Doncaster and Newham) were chosen as pilot sites (termed ‘demonstration sites ’ by the Department of Health). Full details of the clinical services that were developed in the two demonstrations sites and the outcomes they obtained in their first year can be found in Clark *et al.* (2009) and Richards & Suckling (2009).

Briefly, each demonstration site received substantial funds to recruit and deploy an expanded workforce of CBT-focused psychological therapists. Doncaster had been pioneering the use of low-intensity therapies (especially guided self-help) and chose to particularly expand the work force that delivered these treatments, although some additional capacity to deliver high-intensity interventions (face-to-face CBT) was also developed. Many of the guided self-help sessions were delivered over the telephone. As low-intensity interventions and stepped care are not recommended by NICE for PTSD, the Doncaster site excluded this anxiety disorder but encouraged referrals for other anxiety disorders, as well as depression. Newham initially placed greater emphasis on high-intensity CBT, although it also operated a stepped-care model when appropriate, using a newly recruited workforce of low-intensity therapists (subsequently called psychological wellbeing practitioners or PWPs). The low intensity therapies included computerized CBT (cCBT), guided self-help and psycho-educational groups.

In order to determine whether the demonstration sites were able to achieve the outcomes one might expect from the randomized controlled trials that led to NICE's recommendations for the use of psychological treatments in depression and anxiety disorders, both demonstration sites agreed to adopt a session-by-session outcome monitoring system that had demonstrated its worth in achieving high levels of pre/post-treatment data completeness in community samples (Gillespie *et al.*, 2002). At every clinical contact patients were asked to complete simple measures of depression (PHQ-9: Kroenke *et al.*, 2001) and anxious affect (GAD-7: Spitzer *et al.*, 2006). If specific anxiety disorders (for example, agoraphobia, social phobia, OCD, PTSD) were being treated, patients were also encouraged to complete a validated measure of that disorder (for example, the Revised Impact of Events Scale in PTSD: Weiss & Marmar, 1997). This is because the GAD-7 does not cover key features of specific anxiety disorders such as phobic avoidance, compulsive behaviour and intrusive thoughts, images or impulses.

Since the creation of the NHS in 1948, most patients who received specialist psychological therapy had to be referred by their general practitioner (GP), partly to help constrain NHS costs. However, there was some concern that requiring patients to be referred by a GP might be seen as an impediment to access for some members of the community. For this reason, the demonstration sites were allowed to also accept self-referrals as an experiment to see whether it identified people with mental health problems who would not otherwise have access to services.

The main findings from the first year of operation of the two demonstration sites were as follows:

### Clinical problems

The two sites saw somewhat different populations. Although Doncaster did not use formal diagnoses, GP referral letters mentioned depression as the main problem in 95% of cases. In the remaining 5% anxiety was mentioned as the main problem, mainly GAD (3.9%). Newham established *International Classification of Diseases* (ICD-10) diagnoses. Main problems were: depression (46% of patients), anxiety disorders (43%) and other problems (11%).

### Numbers seen

Taken together, the two sites saw an impressively large number of people (over 3,500) in the first year, with the use of low-intensity therapies and stepped care being the key ingredients for managing large numbers. For this reason, as the year progressed the Newham site increased the size of its PWP workforce.

### Data completeness

**Figure 1:**
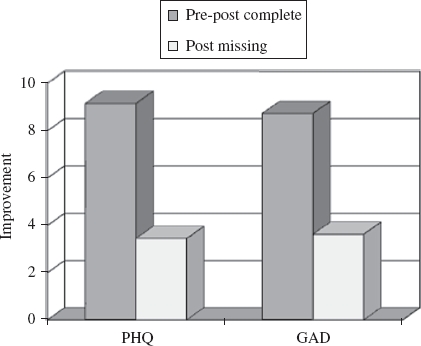
Improvement in PHQ-9 and GAD-7 scores between initial assessment (pre-) and last available session (post-) in people who either completed both the pre- and post-treatment CORE-OM or who failed to complete the CORE-OM at post. Data from the Newham Demonstration site. Figure derived from Clark *et al.* (2009).

The session-by-session outcome monitoring system ensured that almost all (over 99% for Doncaster and 88% for Newham) patients who received at least two sessions had pre- and post-treatment PHQ-9 and GAD-7 scores. For patients who discontinued therapy earlier than expected, the scores from the last available session were used as post-treatment scores. As well as the new session-by-session outcome monitoring scheme, the sites also obtained outcome data on the Clinical Outcomes in Routine Evaluation Outcome Measure (Barkham *et al.*, 2001) using a more conventional pre and post-treatment only data collection protocol. As is usual in community samples, this protocol produced a much lower data completeness rate (6% in Doncaster, 54% in Newham), mainly due to missing post-treatment scores. Figure 1 shows the mean improvements in depression (assessed by the PHQ-9) and anxiety (assessed by the GAD-7) in patients treated in Newham who did, and did not, provide post-treatment data on the conventional (CORE-OM-based) outcome monitoring protocol. Patients who failed to provide post-treatment data in the conventional system showed less than half of the improvement of those who provided post-treatment data (Clark *et al.*, 2009). This leads to the conclusion that services that have substantial missing data rates are likely to overestimate their effectiveness. For this reason, session-by-session outcome monitoring was adopted in the subsequent national roll-out of IAPT (see below).

### Self-referral versus GP referral

Newham, which has a mixed ethnic community, made extensive use of self-referral. Comparisons of self-referred and GP-referred patients indicated that the self-referrers had similarly high PHQ-9 and GAD-7 scores as the GPs', referrals but tended (non-significantly) to have had their problem longer. Importantly, self-referrals more accurately tracked the ethic mix of the community (minorities were under-represented among GP referrals) and had higher rates of PTSD and social phobia, both conditions that traditionally tend to be under-recognized. These findings led the government to include self-referral in the subsequent national roll-out.

### Outcomes

The high level of data completeness on the PHQ-9 and GAD-7 made it possible to accurately assess any clinical improvements that patients achieved while being treated in the demonstration sites. All patients who received at least two sessions (including assessment) were included in the analysis, irrespective of whether they were coded as completers or drop-outs by their therapist. As a group, patients treated in both sites showed meaningful improvements (pre/post-treatment uncontrolled effect sizes of 0.98-1.26). Individuals were considered clinically recovered if they scored above the clinical cut-off on the PHQ and/or the GAD at pre-treatment and below the clinical cut-off on both at post-treatment. Using this criterion, 55% (Newham) and 56% (Doncaster) of patients recovered. Self-referrers and patients from ethnic minorities were no less likely to recover than (respectively) GP referrals and Caucasians.

The economic argument for IAPT (Layard *et al.*, 2007) was based on the assumption that clinical improvement would be sustained and that treatment would improve peoples' employment status as well as symptoms. To assess whether clinical improvements were sustained, patients in both sites were asked to re-complete the outcome measures 9 months (on average) after discharge. Unfortunately, data completeness at follow-up (36% in Newham and 51% in Doncaster) was much lower than at post-treatment (88% and 99% respectively). However, among those people who did provide data, the gains that were achieved in therapy were largely maintained. To assess employment changes, pre-treatment and post-treatment employment status was compared. It had been assumed that IAPT services would achieve an overall improvement in employment status in 4% of the total treated cohort (Layard *et al.*, 2007). The observed rate was 5%.

Although the outcomes observed in the demonstration sites were broadly in line with expectation, it is important to realize that the sites were not set up as randomized controlled trials and it is likely that some of the observed improvement would have happened anyway (for example, natural recovery). Various studies suggest that natural recovery rates over a period of time that is similar to the duration of IAPT treatment are high among recent onset (< 6 months) cases of depression and anxiety disorders but are substantially lower among more chronic cases. Building on this observation, Clark *et al.* (2009) separately computed the recovery rates for recent onset and chronic cases. Most cases (83% in Newham, 66% in Doncaster) had been depressed or anxious for over 6 months and it seemed safe to conclude that treatment had provided added benefit to this group as the recovery rates (52% at each site) comfortably exceeded the 5-20% one might expect from natural recovery or minimal intervention. However, among the minority of cases with a recent onset, it was not possible to exclude the possibility that much of the improvement may have been due to natural recovery (see Clark *et al.*, 2009).

## Description of the national programme

### Initial funding, goals and targets

Following the success of the Newham and Doncaster demonstration sites and the submission of a detailed business case, which included reviews of controlled evaluations of CBT in depression and anxiety disorders, the UK Government announced that it intended to greatly increase the availability of evidence-based psychological therapies for depression and anxiety disorders throughout England through a phased rollout that would last several years. Funding for the first three years was announced: year 1 £33 million, year 2 an additional £70 million on top of the year 1 sum (which had become recurrent), year 3 an additional £70 million on top of the year 1 and 2 sums. Total over 3 years: £309 million.

The funding was allocated to train up to 3,600 new psychological therapists (60% high-intensity CBT therapists, 40% PWPs) and to deploy them, along with existing experienced clinicians, in new psychological treatment services for depression and anxiety disorders that operate on stepped-care principles. The training programme initially focused on CBT, as (1) it is recommended by NICE for both depression and anxiety disorders, and (2) it is the therapy where the manpower shortage was considered to be greatest.

Targets were set for the number of patients that would be seen by the services in the first three years and there was an expectation that 50% would ‘move to recovery ’ in terms of their symptomatology. In addition, it was expected that 25,000 fewer people would be on sick pay or receiving state benefits. At least 20 of England's 154 PCTs were expected to establish new ‘IAPT ’ services during the first year (2008/09), with further PCTs joining in future years.

In order to realize these goals, the Department of Health established a series of expert groups that helped devise the necessary training programme and specified key features of the IAPT clinical services. A large number of documents providing guidance to courses and PCTs were produced, most of which can be viewed on the IAPT website (http://www.iapt.nhs.uk). Table II lists the key documents, including the national *IAPT Implementation Plan* (Department of Health, 2008).

**Table II:** Key IAPT reference documents (available at http://www.iapt.nhs.uk) with publication dates in parentheses, when relevant.

*IAPT Implementation Plan: National Guidelines for Regional Delivery* (February 2008)
*IAPT Implementation Plan: Curriculum for High-Intensity Workers*
*IAPT Implementation Plan: Curriculum for Low-Intensity Workers*
*IAPT Impact Assessment* (February 2008)
*IAPT Equality Impact Assessment* (February 2008)
*IAPT Supervision Guidance*
*IAPT Commissioning Toolkit* (April 2008)
*Realising the Benefi ts: IAPT at Full Roll-Out* (February 2010)
*The Operating Framework for the NHS in England 2011/12*
*No Health Without Mental Health* (February 2011)
*Talking Therapies: A Four Year Plan* (February 2011)
*Which Talking Therapy for Depression?* (March 2011)
*Commissioning Talking Therapies for 2011/12* (March 2011)
*IAPT Data Handbook 2* (June 2011)

During the first two years, all funds were held centrally by the Department of Health and distributed through England's ten strategic health authorities (SHAs), who commissioned appropriate regional training courses and selected the PCTs that would receive the new trainees and other resources needed to set up a new IAPT service. Rather than place a few trainees to each PCT, it was decided to initially allocate a substantial number of trainees to a few PCTs (early adopters) who would then have the resources to create a service with sufficient capacity ensure patients are seen promptly. During the third year, the principle for distributing funds changed and much of the money for IAPT went into the general bundle of funds that PCTs receive to finance all of their healthcare work.

### Training

In order to guide the training of the new workforce, the Department of Health commissioned and distributed separate national curricula for the training of high-intensity CBT therapists and PWPs. As the main aim of the IAPT programme is to increase the availability of treatments recommended by NICE, the high-intensity CBT curriculum is closely aligned to the particular CBT programmes that had been shown to be effective in the RCTs that contributed to NICE's recommendations. A wide range of general CBT assessment and intervention strategies are included in the curriculum. In addition, trainees are required to be taught at least two evidence-based treatments for depression (cognitive therapy and behavioural activation) and at least one specific, evidence-based treatment for each anxiety disorder. In panic disorder, examples include Barlow and colleagues' CBT programme (Barlow and Craske, 2007) and Clark and colleagues' cognitive therapy programme (Clark and Salkovskis, in press). In PTSD, examples include Foa's imaginal reliving (Foa & Rothbaum, 1998), Ehlers and Clark's cognitive therapy (Ehlers & Clark, 2000; Clark & Ehlers, 2004), and Resick's cognitive processing therapy (Resick *et al.*, 2007). Roth and Pilling (2008) developed a competency framework for many of the leading empirically supported CBT treatments for depression and anxiety disorders, and the high-intensity curriculum aims to ensure that these are covered in IAPT training programmes. In addition to specifying the skills that trainees should acquire, the curriculum also specifies how these skills should be assessed (through a mixture of ratings of actual therapy sessions using the revised version of the Cognitive Therapy Rating Scale (CTS-R) (Blackburn *et al.*, 2001) and written assignments in the form of case reports and essays).

A separate curriculum was issued for PWP training. The four sections of the curriculum cover: (1) engagement and assessment, (2) evidence-based low intensity treatments, (3) values, policy, culture and diversity, (4) working within an employment, social and healthcare context. As low-intensity working is relatively new, there are few published therapist manuals. To redress this shortfall, a substantial set of teaching aids developed by David Richards (one of the pioneers of low-intensity work) and his colleagues were produced to supplement the curriculum. As with the high-intensity curriculum, assessment procedures are also specified, with particular emphasis being placed on structured role-plays covering a wide range of different skills.

Both the high-intensity CBT and the PWP training programmes are conceived as joint university and in-service training. Over a period of approximately 1 year high-intensity trainees attend a university-based course for lectures, workshops and case supervision two days a week, while PWPs attend university for one day per week. For the rest of their time, both sets of trainees work in an IAPT service where they receive further regular supervision. The services are also encouraged to provide the trainees with the opportunity of directly observing therapy sessions conducted by experienced staff who work in the service.

### IAPT service model

A general framework for IAPT services was outlined in the national *Implementation Plan* (Department of Health, 2008). The framework specifies several key principles for the operation of the services while leaving considerable scope for local determination. The key principles include:

- Access to the service through self-referral as well as referral by general practitioner.
- A person-centred assessment that identifies the key problems that require treatment and their social and personal context. Goals for therapy are identified and a treatment plan is jointly agreed.
- Stepped care in which many people with mild to moderate depression or anxiety disorders are offered treatment with a PWP initially. Many people recover with such treatment. Individuals who do not should be offered a further course of high intensity treatment. For people with more severe depression or anxiety and for everyone with PTSD, immediate high-intensity treatment is recommended. All treatments that are offered should be in line with NICE recommendations.
- Access to an employment adviser if employment (lack of, or danger of losing) is an issue. Services are encouraged to involve employment advisers in treatment plans from the very beginning as making progress with employment issues can greatly facilitate psychological recovery and visa versa.
- Use of the IAPT minimum dataset (see *IAPT Data Handbook 2* for full details: Department of Health 2011c). This includes giving the PHQ-9 and GAD-7 every session along with some other patient self-report measures that focus on specific anxiety disorders, when these are relevant. All data is entered into an electronic database that enables therapists and their supervisors to monitor patients' progress and adjust treatment plans, if required.
- All therapists should receive weekly outcome-informed supervision which ensures that all cases are discussed at regular intervals and decisions about step-up/step-down are made in a timely fashion (see *IAPT Supervision Guidance).*
- Because of the importance of obtaining outcome data on almost all patients who receive treatment, the services are asked to ensure that at least 90% of patients who are seen at least twice in a service have a pre-treatment and post-treatment (or last available session) score on the main outcome measures. For patients who exceed the clinical cut-off for depression and/or anxiety at pre-treatment, ‘recovery ’ is operationalized as moving to below the clinical cut-off for both depression and anxiety at post-treatment.

## Progress to date

At the time of writing (Spring 2011), the IAPT programme is midway through its third year. Progress to date includes:

- IAPT services have been established in 95% of PCTs. However, there is wide variation in the number of therapists employed in the services and, as a consequence, they vary substantially in the number of patients that they are able to see. It is therefore calculated that only around 60% of the population has access to an IAPT service. For this reason, there is a need to further expand the services in coming years (see later section on future developments).
- Over 3,660 new high-intensity therapists and PWPs have been appointed and will have completed their training by the end of the year.
- The IAPT services are currently seeing around 310,000 patients per annum and aim to see around 900,000 per annum by 2015 when the roll-out of the programme should be complete.
- National data collected at the end of the second year of the programme showed that it is on target in terms of the number of people seen (399,460 compared to a target of 400,000), the number of people who have moved off sick pay and/or state benefits (13,962 compared to a target of 11,100) and has recovery rates which are approaching expectation (an average of 40% compared to a target of 50%).

## Lessons from the first phase of the implementation

In addition to the broad performance figures given above, the Department of Health has released two reports that provide more detailed analysis of the national IAPT programme during its first year of operation (1 October 2008 to 30 September 2009). During this period 35 PCTs established an IAPT service, 32 of whom provided data for analysis.

The first report (Glover *et al.*, 2010) particularly focused on issues to do with equity of access, descriptions of the treatments offered, and overall outcome. With respect to equity of access, both genders were fairly represented in the year one IAPT services. The most recent Adult Psychiatric Morbidity Survey (McManus *et al.*, 2007) shows that 61% of people in the community with a common mental disorder are female, which was very similar to the rate in IAPT services (66% female). However, people over 65 years old and people from black and minority ethnic (BME) groups were somewhat underrepresented. Part of the reason for the latter finding may have been the slow development of a self-referral route into the services. Clark *et al.* (2009) found that self-referral produces a more equitable pattern of access for different ethnic groups but only 10% of patients came through self-referral (compared to 21% in the Newham demonstration site). Looking at clinical conditions, it was difficult to assess equity of access accurately as for 39% of patients an ICD diagnosis was not recorded. However, among the 61% for whom diagnoses were recorded, there was an over-representation of patients with depression or mixed anxiety and depressive disorder (MADD), compared to prevalence rates found in epidemiological studies. There was also under-representation of patients with persistent anxiety disorders, such as PTSD, OCD, panic disorder, social phobia and agoraphobia, as less than 10% of patients had these diagnoses, whereas around a third of patients should have these disorders if access was equitable (see McManus *et al.*, 2009).

The first report also found that the majority of patients received NICE-compliant treatment. The NICE-recommended low-intensity interventions that were provided included guided self-help, psycho-education groups, behavioural activation, cCBT and structured exercise. NICE-recommends CBT as a high-intensity psychological therapy for depression and for all the anxiety disorders that are currently covered by guidelines. In line with this recommendation, almost everyone with a recorded diagnosis of social phobia, specific phobia, agoraphobia, or OCD received CBT. For patients with a recorded diagnosis of GAD or PTSD, CBT was also the most commonly provided treatment. However, a significant number of patients received counselling, which is not recommended by NICE for these conditions. For patients with a recorded diagnosis of depression, CBT and counselling were equally likely to be offered and both are recommended by NICE, although counselling has a more restricted recommendation in terms of the range of cases for which it is considered relevant (see Table I). Turning to clinical outcomes, a recovery rate of 42% was observed among suitable patients who were likely to have received at least some treatment (defined as having at least two sessions on the assumption that the first session was always assessment) . However, there was considerable variability in recovery rates between sites.

The second report (Gyani *et al.*, 2011) explored the observed variability in recovery rates in further detail in order to identify site and other characteristics that were associated with higher recovery rates. The analyses focused on patients who were clinical cases on entry into the service, had received at least two sessions and had completed their involvement with the services. Pre- to post-treatment data completeness for these patients was good (> 90%). The findings, which are briefly summarized below, generally support the IAPT clinical model and highlight the value of following NICE guidelines.

Patients had a higher chance of meeting recovery criteria if they were treated at sites that had the following characteristics:

- Higher step-up rates from low-intensity to high-intensity therapy among those who had failed to respond adequately to the former (i.e. the services were making good use of stepped care).
- Higher average numbers of therapy sessions at low intensity and at high-intensity (highlighting the importance of providing an adequate dose of treatment).

Although most patients received NICE recommended treatments, for some clinical conditions a significant minority of patients received a treatment not recommended by NICE. This created a natural experiment in which it was possible to assess whether deviation from NICE recommendations was associated with a reduction in recovery rates. One of the natural experiments concerned the contrast between CBT and counselling. For depression NICE recommends both treatments for mild to moderate cases. Consistent with this recommendation, there was no difference in the recovery rates associated with CBT and counselling among patients with a diagnosis of depression. In contrast to the recommendations for depression, NICE does not recommend counselling for the treatment of GAD. Consistent with this position, CBT was associated with a higher recovery rate than counselling among patients with a diagnosis of GAD. A further natural experiment concerned the contrast between guided self-help and pure (non-guided) self-help. NICE only recommends guided self-help in depression. Consistent with this position, guided self-help was associated with a higher recovery rate than pure self-help among patients with a diagnosis of depression. Taken together, these findings would appear to support the value of aligning clinical interventions with NICE guidance. However, this conclusion needs to be treated with caution as these ‘natural experiments ’ are not randomized clinical trials.

A final variable considered in the second report was initial severity. Patients with higher initial depression or anxiety scores were less likely to meet recovery criteria (dropping below the clinical/non-clinical threshold) at the end of treatment, but their overall amount of symptomatic improvement was at least as large as that observed in milder cases. This suggests that the IAPT services are beneficial for individuals with a wide range of symptom severity.

## Future development of the programme

Following the success of the first three years of the IAPT programme, the government announced in February 2011 a further NHS investment of £400 million to complete and extend the programme over the period 2011-2015. Full details of the next phase can be found in the mental health policy entitled *No Health Without Mental Health* (Department of Health, 2011a) and in the accompanying document entitled *Talking Therapies: Four Year Plan of Action* (Department of Health, 2011b).

Briefly, a major component of the next phase is completion of the roll-out of IAPT services for adults. This will require the training of a further 2,400 new high-intensity and PWP therapists. At the same time, continuing professional development (CPD) short courses will be used to further enhance and update existing clinicians' skills in non-CBT therapies that are recommended by NICE for the treatment of mild to moderate depression, in order to widen patient choice for evidence-based treatments within IAPT services. The CPD courses are aligned to national curricula and published competencies (available at www.iapt.nhs.uk) and cover interpersonal psychotherapy, couples therapy, a form of brief psychodynamic therapy (dynamic interpersonal therapy) and counselling.

A challenge for the completion of the programme is a change in the way in which the funding for the training and new posts will be managed. In the first two years of the programme, all funds were centrally held and ring-fenced. It was therefore possible to ensure that they were exclusively spent on the IAPT workforce. In year three (2010/11) a significant proportion of the funds were allocated within general NHS budgets (technically termed ‘PCT baseline funding ’) as are the funds for most mainstream NHS activities. Unfortunately, there is evidence that some of this money was not spent on IAPT, although the numbers of new trainees in that year remained on target. In the next phase, almost all funds will be allocated within general NHS training and PCT budgets and there is a risk that some geographical areas will invest less in IAPT than expected. To mitigate this risk, the Department of Health has specifically highlighted the importance of IAPT by including it for the first time in *The Operating Framework for the NHS* (Department of Health, 2010). To assist local commissioners in their decision making, a guidance document that highlights the value of extending IAPT has been issued. *Commissioning Talking Therapies for 2011/12* (Department of Health, 2011d) outlines the major savings in other costs to the NHS and to society that can be realized by increasing the availability of evidence-based psychological treatments for depression and anxiety disorders. One of the NHS savings relates to the medical treatment of chronic physical health problems, such as coronary heart disease, obstructive pulmonary disease and diabetes, all of which are more costly to medically manage when a person is also depressed.

A further challenge concerns the relationship between IAPT and other NHS mental health services. The decision to deploy the IAPT workforce in new services was important in order to ensure consistency of the training experience and clinical supervision, compliance to NICE guidance, and high levels of data completeness. However, it is also important that the new services are well integrated with other NHS provision for mental health problems. For this to happen, local areas need to develop coherent care pathways that provide clarity about who should be seen, by which service, at which point in their care. Transition between services should be facilitated, whenever it is appropriate. It is essential that commissioners understand what their local IAPT service can, and cannot, offer when considering any reorganization of other services so they do not inadvertently reduce provision for individuals with some conditions or complexities whose care is best provided elsewhere.

Reporting on the performance of IAPT services will also be enhanced in the next phase in order to provide clinicians with valuable information that they can use to further develop the accessibility and effectiveness of their IAPT services, as well as increasing transparency for commissioners and the public.

A new feature of the next phase will be the creation of a version of the IAPT programme for children and young people. Many of the anxiety disorders that are seen in adult services start in adolescence or earlier and can severely interfere with social and educational development. For this reason it is important to make effective psychological treatments for these conditions, as well as other mental health problems, available in childhood and adolescence. The under-representation of people over 65 and people from BME communities that was evident in some IAPT services in the first phase of the programme will also be addressed by initiatives that focus on these individuals.

## Conclusions

England is midway through the development of a large-scale programme that aims to greatly increase the availability in the NHS of NICE-recommended psychological therapies for depression and anxiety disorders. Following successful pilot work in Doncaster and Newham, a phased national roll-out was planned and is processing broadly in line with expectation. Training of the new workforce has been closely aligned to the skills and competencies required for the specific treatments recommended by NICE and a session-by-session outcome monitoring system has ensured unprecedentedly high levels of pre- to post-treatment data completeness for key outcome measures. Large numbers of people who would not previously have had the option of a psychological treatment have accessed the services. Average recovery rates are approaching, but are not yet at, those expected from the randomized controlled trials that generated the NICE recommendations. As expected, gains in terms of employment and reductions in state benefits have also been observed. Lessons from the early phases of the programme suggest ways in which less well performing services may evolve to achieve the outcomes shown by the best services (which are in line with, or exceed expectation). In the meantime, the extremely high levels of data completeness achieved by IAPT has brought greater transparency to mental health services and helped clinicians and commissioners to identify both areas of excellence and areas that require further attention as the NHS strives to further improve the care it offers people with depression and anxiety disorders.
